# Framing vs. supporting evidence in L2 argumentative writing: a mixed-methods study of Chinese EFL learners

**DOI:** 10.3389/fpsyg.2025.1705232

**Published:** 2025-11-26

**Authors:** Rui Yang

**Affiliations:** 1 School of Foreign Languages, Anhui Science and Technology University, Chuzhou, Anhui, China; 2 Key Laboratory of Human–AI Collaborative Translation and Education, Anhui Science and Technology University, Chuzhou, Anhui, China

**Keywords:** L2 argumentative writing, evidence use, framing evidence, supporting evidence, proficiency differences, source-based writing, mixed methods, assessment

## Abstract

**Background:**

Evidence integration is central to argumentative writing, yet the relationships between different evidence functions and L2 writing quality across proficiency levels remain under-examined.

**Methods:**

Using an explanatory sequential mixed-methods design (QUAN → qual), we analyzed 542 classroom, timed English argumentative essays by Chinese undergraduates (30 min; 120–180 words). Texts were functionally coded as framing evidence (constructing the inferential scaffold) and supporting evidence (verifiable data, examples, expert attribution, etc.). Inter-rater reliability for the evidence scheme was high; writing quality was represented by standardized rubric scores. We ran ordinary least squares (OLS) regressions stratified by proficiency (high/mid/low) to test whether evidence type predicted scores, and used qualitative close readings to illustrate typical evidence–reason coupling.

**Results:**

Overall, framing evidence predominated. The mid-proficiency group showed the most balanced framing–supporting configuration; the low-proficiency group was weak on both types of evidence. Stratified regressions indicated that only in the mid-proficiency group did evidence type significantly predict writing scores (*β* ≈ 0.40, 95% CI ≈ 0.19–0.62); effects in other groups were not robust, and model fit was modest (low–moderate R^2^).

**Conclusion:**

The findings suggest a developmental shift from “having evidence” to “using evidence well.” Once writers can supply basic evidence, further gains in quality hinge less on adding types or quantity and more on selecting precise evidence, explaining it clearly, and aligning it tightly with the claim—that is, achieving functional fit and linking through explicit warrants. Instruction and assessment should therefore pivot from “whether/how much evidence” to how evidence is selected, interpreted, and embedded in the inferential chain.

## 1 Introduction

At the heart of argumentative writing lies the task of “supporting defensible claims with appropriate evidence” (Cottrell, 2011; Brem and Rips, 2000). Recent systematic reviews identify L2 argumentative writing as a growing research focus, centering on the interplay among argument structure, instructional interventions, and assessment, and calling for more fine-grained accounts of the role of evidence in argumentation (Amini Farsani et al., 2025). In integrated reading–writing assessment contexts, studies show that *how* evidence is integrated—rather than merely *whether* it is cited—more strongly relates to argumentative effectiveness (e.g., coherence, quality of reasoning) (Chuang and Yan, 2023). Rater-experiment work further indicates that both the amount and quality of evidence significantly shape judgments on the “argumentation” dimension (Chuang, 2025; Nussbaum et al., 2019). From a process perspective, keystroke-logging research reveals elevated cognitive load and distinct process trajectories when students generate key elements such as claims, data, and rebuttals, underscoring the need for more explicit genre and argumentation scaffolds (Tian et al., 2024). In addition, intervention evidence grounded in dynamic assessment suggests that diagnostic-mediation cycles can raise learners’ developmental levels in source integration and argumentation (Yu and Poehner, 2025). Against this backdrop, the present study focuses on Chinese EFL learners, operationally distinguishing *framing evidence* and *supporting evidence*, comparing their distributions across proficiency groups (high/mid/low), and testing the relationships and effect sizes linking different types of evidence to writing scores. In doing so, the study provides actionable variables and empirical grounding for measuring and teaching argumentation (Peltzer et al., 2024a, 2024b; Peltzer et al., 2025; Burnell et al., 2023; Panadero, 2025; Bin Dahmash, 2025).

## 2 Literature review

### 2.1 The importance of evidence in L2 argumentative writing

In L2 argumentative writing, evidence functions as the “hub” connecting claims to chains of reasoning; its presentation and quality are closely associated with argumentative effectiveness and with raters’ judgments (Chuang and Yan, 2023; Chuang, 2025). Explicit instruction built on Toulmin-type models can significantly increase students’ production of key argumentative elements (e.g., claims, evidence/data, rebuttals) and improve overall argumentative structure and quality (Yang, 2022; Yang and Pan, 2023; Samad et al., 2024). At the feedback level, structured approaches such as *rubrics plus model texts (exemplars)* have been shown to improve L2 writing performance; thanks to their standardization and replicability, such feedback is especially feasible and scalable in large classes (Peltzer et al., 2024a, 2024b; Winstone and Carless, 2020; York University Teaching Commons, 2021; Burnell et al., 2023; Panadero, 2025; Bin Dahmash, 2025). Process evidence further indicates that different argumentative elements (claims, evidence, rebuttals/qualifiers) entail distinct processing loads and behavioral patterns, suggesting that instruction and assessment should explicitly differentiate the functions of evidence (Tian et al., 2024; Cottrell, 2011). Because proficiency-related differences co-vary with linguistic resources (syntactic and lexical complexity), which may interact with evidence use to influence writing quality, studies of the evidence–quality link should control for language-ability background (Atak and Saricaoglu, 2021; Saricaoglu and Atak, 2022; McCann, 1989).

### 2.2 Research on “evidence” in L2 argumentative writing

Work on integrated reading–writing tasks consistently finds that how evidence is selected, interpreted, and integrated—i.e., its alignment with claims/task purposes and its accuracy—is closely tied to argumentative quality and can indirectly affect writing scores via argumentative structure; by contrast, sheer citation counts do not necessarily yield higher scores (Chuang and Yan, 2023; Plakans and Gebril, 2013; Chuang, 2025; Brem and Rips, 2000). From a structural perspective, Qin and Karabacak (2010), applying a Toulmin framework to Chinese university EFL essays, report low rates of rebuttal-related elements and predominantly implicit warrants, highlighting a weakness in the functional linkage between evidence and claims. This structural shortcoming echoes Wingate’s (2012) finding in undergraduate contexts: students and teachers often lack a clear concept of *argument*, and courses frequently fail to make “argument-organized writing” explicit; consequently, students struggle to transform evidence into expressions that serve claims and reasoning. From a washback perspective, Liu and Stapleton (2014) note that test-oriented training can suppress attention to counter-positions and rebuttal, thereby constraining the textual realization of critical thinking.

Two primary strands characterize the literature. A *product-oriented* strand annotates the correspondences between evidence and claims/counterarguments in student texts and then compares groups by proficiency or prompt (Plakans and Gebril, 2012; Chuang and Yan, 2023). A *process-oriented* strand uses reading-writing traces, keystroke logs, and screen recordings to track how learners search for sources, select, paraphrase, and merge information into argumentation, revealing substantial cognitive demands and wide strategy variation (McCulloch, 2013; Wette, 2018). With increasing proficiency, direct copying declines and paraphrasing improves; yet learners still falter in “source-attribution conventions” and in *functionally integrating* information to support claims, signaling a continued need for explicit, actionable training (Keck, 2014; Wette, 2018; see also Wingate, 2012; Judd et al., 2006).

### 2.3 Framing evidence and supporting evidence

In argumentation research, evidence is typically understood as a holistic resource that supports claims; its sources include both learners’ schematic knowledge and external texts/materials (Sandoval and Millwood, 2005). To capture functional differences among data types used to support claims, Packer and Timpane (1997) classify evidence into seven categories: expert opinions, statistical data, examples, personal experience, common sense, logical analysis, and analogy. Building on this typology, Zhang (2018) proposes two higher-order categories: *factual evidence*, characterized by objective verifiability and often treated as “hard evidence,” and *non-factual evidence*, which relies primarily on subjective judgment or indirect inference and is often regarded as “soft evidence.” This binary framework, centered on verifiability, provides a clear analytic path for assessing the validity and applicability of different kinds of evidence in argumentation.

From a functional perspective, evidence can be further divided into two types: (1) *explanatory evidence*, which elaborates and specifies the object of discussion by clarifying background, conditions, and mechanisms; and (2) *justificatory/persuasive evidence*, which leads readers to accept the author’s claim through sufficiently warranted reasoning. Together they form a continuum from “explanatory” to “empirical.” In general, *framing evidence* emphasizes providing explanatory, structure-building scaffolds for reasoning, whereas *supporting evidence* focuses on justifying claims with verifiable materials. Their typical synergy is: first, use framing evidence to establish the argumentative structure and premises; then, use supporting evidence to supply checkable materials, thereby jointly reinforcing the central claim (Brem and Rips, 2000). Accordingly, what Du and List (2021) term *transformed evidence*—reorganizing textual information with prior knowledge or other sources in novel ways—can be viewed as a prototypical realization of framing evidence.

By contrast, *supporting evidence* remains relatively independent of the subclaim at the discourse level and typically takes the form of verifiable factual materials, such as representative cases, statistics and figures, authoritative quotations, or research findings. Its core function is to provide a checkable evidential basis for subclaims and thereby enhance the persuasive force of the central claim.

In sum, although prior work suggests that *how* evidence is integrated better explains argumentative quality than *whether* it is cited, there remains a lack of operationalized, systematic comparisons—under timed, source-integration conditions—of the distributions and relative contributions of *framing evidence* and *supporting evidence* across proficiency levels. To address this gap, the present study operationally distinguishes these two types of evidence in a Chinese EFL sample and, controlling for background variables such as linguistic complexity, examines how their functional alignment relates to writing scores.

Research Questions

1. What are the distributions of evidence types in English argumentative essays produced by university students at different proficiency levels? Specifically, what proportions do *supporting evidence* and *framing evidence* account for?
2. Do different types of evidence in English argumentative essays produced by students at different proficiency levels affect essay quality?

## 3 Methods

This study examines the distributional features of evidence types and their relationships with writing quality under unified genre and task conditions in timed, in-class argumentative writing by Chinese university students, and compares differences across proficiency levels. We adopted an explanatory sequential mixed-methods design (QUAN → qual; Creswell and Creswell, 2018): in Stage 1, we conducted quantitative coding to produce descriptive statistics and relational analyses; in Stage 2, we drew purposive samples based on the quantitative results and performed close textual analysis to validate and refine the *supporting–framing evidence* framework, thereby generating actionable evidence for pedagogy and assessment. The overall design is cross-sectional; the present report constitutes a sub-study within a larger mixed-methods program.

### 3.1 Participants

Participants were first-year undergraduates from five institutions (four in Northeast China and one in Tianjin). The institutional spectrum covered “985” universities, provincial universities, and vocational colleges, increasing social and linguistic diversity in the sample. We collected 556 essays; after removing 14 incomplete/invalid scripts, the valid sample comprised 542 essays. Gender: 173 male (32%) and 369 female (68%); age 18–21. Majors spanned engineering, business/management, and teacher-education programs (e.g., Vehicle Engineering, Industrial Design, Energy and Power, Mechanical Design and Automation, Engineering Management, Bioengineering, Economics, International Trade, Human Resources, Information Systems, Hospitality and Tourism, Tourism Management, Early Childhood Education, Culinary Arts).

### 3.2 Writing task

Under proctored classroom conditions, participants completed a timed English argumentative essay (30 min; 120–180 words). Administration was paper-and-pencil with uniform prompts and time limits; any external assistance (including peer/teacher guidance, online polishing services, or generative tools) was explicitly prohibited. The prompt was: “Is it right for marine parks to stay open?” This context aligns with college English writing instruction and CET-4 preparation, facilitating concentrated sampling and enhancing the authenticity, comparability, and replicability of the corpus.

### 3.3 Variables and measures

#### 3.3.1 Outcome variable (writing quality)

The dependent variable was the total essay score, rated according to the official CET-4 rubric. CET-4 was chosen because its scoring dimensions (content/organization, language, coherence/logic) align with university-level writing constructs and offer standardization and cross-case comparability; the total score is continuous, enabling statistical analysis. Two raters independently and blindly scored each essay; the mean was used. If the inter-rater difference exceeded a preset threshold (3 points), a third party (the present author) reviewed and adjudicated.

#### 3.3.2 Predictor variable (types of evidence)

Drawing on Zarefsky (2019) and Hornikx (2008), and incorporating Du and List’s (2021) surface–deep distinction, evidence was classified into two levels and six categories:

Supporting evidence (surface level):

1. *Statistical data* (proportions, trends, quantitative comparisons);
2. *Established facts/shared beliefs* (widely accepted knowledge statements);
3. *Expert opinion* (attributed quotations or paraphrases indicating source/identity);
4. *Personal experience* (first-hand experiences and observations);
5. *Examples/cases* (fact-based instances in specific situations, including micro-cases).

Framing evidence (deep level):

1. *Reason reconstruction/frame-setting* (delineating problem boundaries, setting evaluation criteria, and building causal or value-judgment frameworks that organically link subclaims with evidence).

#### 3.3.3 Coding unit and derived measures

The coding unit was the Minimum Evidence Unit (MEU), defined by two simultaneous conditions: (a) it contains informational content with source cues (explicit or implicit); and (b) it bears an explicit or inferable linkage to the current subclaim. If a single sentence involves distinct sources or inferential paths, each is counted separately; verbatim repetition or near-synonymous restatement is not additionally counted. This operationalization synthesizes literature on integrated writing and argument coding (see Plakans and Gebril, 2012, 2013; Qin and Karabacak, 2010; Du and List, 2021; Keck, 2014).

### 3.4 Coding procedure and reliability

**Figure 1:**
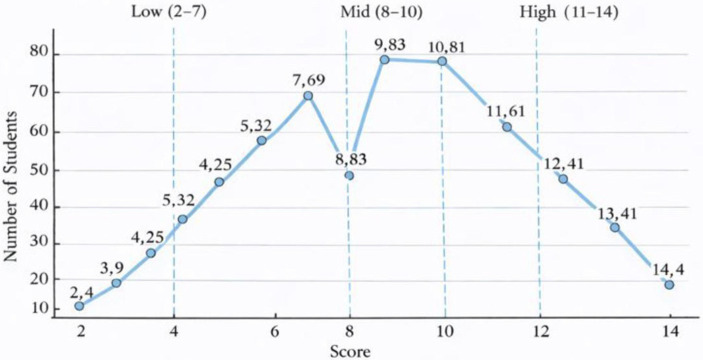
Distribution of English argumentative essay scores.

Prior to full scoring, we calibrated against the CET-4 rubric: two raters independently and blindly scored a random set of 10 essays; the intraclass correlation coefficient [two-way random effects, absolute agreement, single measure; ICC(2,1)] was 0.77. We then discussed discrepant cases and aligned standards. During operational scoring, two raters independently scored in blind conditions and their mean was used; after all scripts were completed, ICC improved to 0.85, indicating good reliability. Grouping was based on the empirical score distribution (see Figure 1) rather than tertiles by headcount: fixed cut-points followed natural breakpoints—Low = 2–7 (*n* = 191), Mid = 8–10 (*n* = 222), High = 11–14 (*n* = 129), total N = 542. This scheme aligns with the unimodal distribution (peak at 9–10) and reduces biases introduced by handling boundary scores.

Two raters received training on the operational definitions of evidence types (see Table 1). They then trial-coded a random set of 10 scripts; discrepancies were discussed and resolved before formal coding commenced. Category agreement was assessed using Cohen’s *κ* (or Krippendorff’s *α*); the adjudicated result was κ = 0.81, indicating high consistency. The workflow proceeded as follows: timed writing was assigned and collected in class; texts were de-identified, numbered, and entered, with cross-checks by two researchers; evidence types were annotated according to *a priori* criteria with spot audits; blind scoring was completed and inter-rater agreement computed; variables were aggregated and data were cleaned following a pre-specified pipeline (including handling of missing values and outliers); statistical analyses were then conducted, and qualitative cases were sampled based on the quantitative results.

**Table 1:** Evidence types and illustrative examples in L2 argumentative writing.

Category	Evidence type	Definition	Example
Supporting evidence^SE^	Statistical evidence^STAT^	Quantitative statements of numbers or measurements; usually independently verifiable.	In a comparative study of wild vs. marine-park dolphins conducted by pre-trainers, captive dolphins showed ≈30% shorter average lifespans than wild dolphins.
Supporting evidence^se^	Established facts and shared beliefs^EFSB^	Background facts or consensual knowledge widely regarded as accurate.	Marine mammals breathe air and nurse their young; studies conducted at marine parks further document these shared physiological needs.
Supporting evidence^SE^	Expert Opinion Evidence^EO^	Statements by knowledgeable authorities or widely recognized figures that provide professional or ethical reasoning.	As Jacques Cousteau noted, “Protecting marine animals is not just an option; it’s our moral obligation.”
Supporting evidence^SE^	Personal experience evidence^PE^	The writer’s first-hand experiences or observations.	When I was in primary school, my parents often took me to marine parks, which made me question how performing affects animal well-being.
Supporting evidence^SE^	Example-based evidence^EX^	Support through specific illustrations or case examples.	For example, structured shows in marine parks can help visitors learn more about sea animals and why protecting the ocean matters.
Framing evidence^FE^	Interpretation or Restatement of reasons^INT^	Further elaboration or reinterpretation that clarifies criteria, mechanisms, or conditions linking evidence to the claim (i.e., makes the warrant visible).	Excessive daily performances can create chronic stress and fatigue in dolphins and whales; therefore, such routines risk long-term harm to animal welfare.

SE, Supporting Evidence; FE, Framing Evidence; STAT, Statistical Evidence; EFSB, Established Facts and Shared Beliefs; EO, Expert Opinion; PE, Personal Experience; EX, Example Based; INT, Interpretation/Restatement. Examples are illustrative for coding purposes and do not assert empirical facts.

### 3.5 Quantitative data analysis

All analyses were conducted in IBM SPSS (Version 26). Addressing the research questions, we fitted multiple/hierarchical regression models and reported standardized coefficients *β* (direction and magnitude), R^2^/ΔR^2^ (variance explained and incremental change), and *p*-values, along with 95% confidence intervals. Statistical significance was set at α = 0.05 (two-tailed), i.e., *p* < 0.05 was taken as statistically significant.

### 3.6 Qualitative phase

We adopted purposive sampling: drawing on group- and pattern-level differences identified in the quantitative phase (proficiency: high/mid/low; strength of *framing evidence* use: strong/weak), we selected cases that balanced maximum variation with typicality. The data source consisted of the original classroom essays. Qualitative analysis employed close textual description: at the paragraph–sentence level, we annotated and reconstructed the “claim–reason–evidence–(frame)” chain with reference to the Toulmin model, focusing on the construction mechanisms of *framing evidence* and its points of articulation with *supporting evidence* (e.g., “frame-first → evidence-later,” “evidence-driven reframing”).

## 4 Results

### 4.1 Distribution of argumentative elements across proficiency levels

Overall, the high-proficiency group performed best in terms of evidence types; the mid-proficiency group showed a slight decline; and the low-proficiency group displayed a substantially lower mean writing score and the lowest mean number of evidence types. Accordingly, Table 2 indicates an uneven pattern across groups in the quantity of argumentative elements presented.

**Table 2:** Writing score and mean number of evidence types across proficiency groups.

Group	n	Writing score (M)	Evidence types (M)
High-proficiency	129	11.77	1.13
Mid-proficiency	222	9.10	1.10
Low-proficiency	191	5.71	0.86

M, mean; n, number of essays. ‘Writing score’ refers to the task score; ‘Evidence types’ refers to the mean count of distinct evidence types per essay.

Table 2 shows that the essay scores for the high-, mid-, and low-proficiency groups (*n* = 129, 222, 191) are 11.77, 9.10, and 5.71, respectively, exhibiting a clear downward trend. The mean number of evidence types likewise decreases with score: 1.13 in the high group and 1.10 in the mid group (comparable), but only 0.86 in the low group—substantially fewer—indicating that the richness of evidence types declines with writing quality.

**Figure 2:**
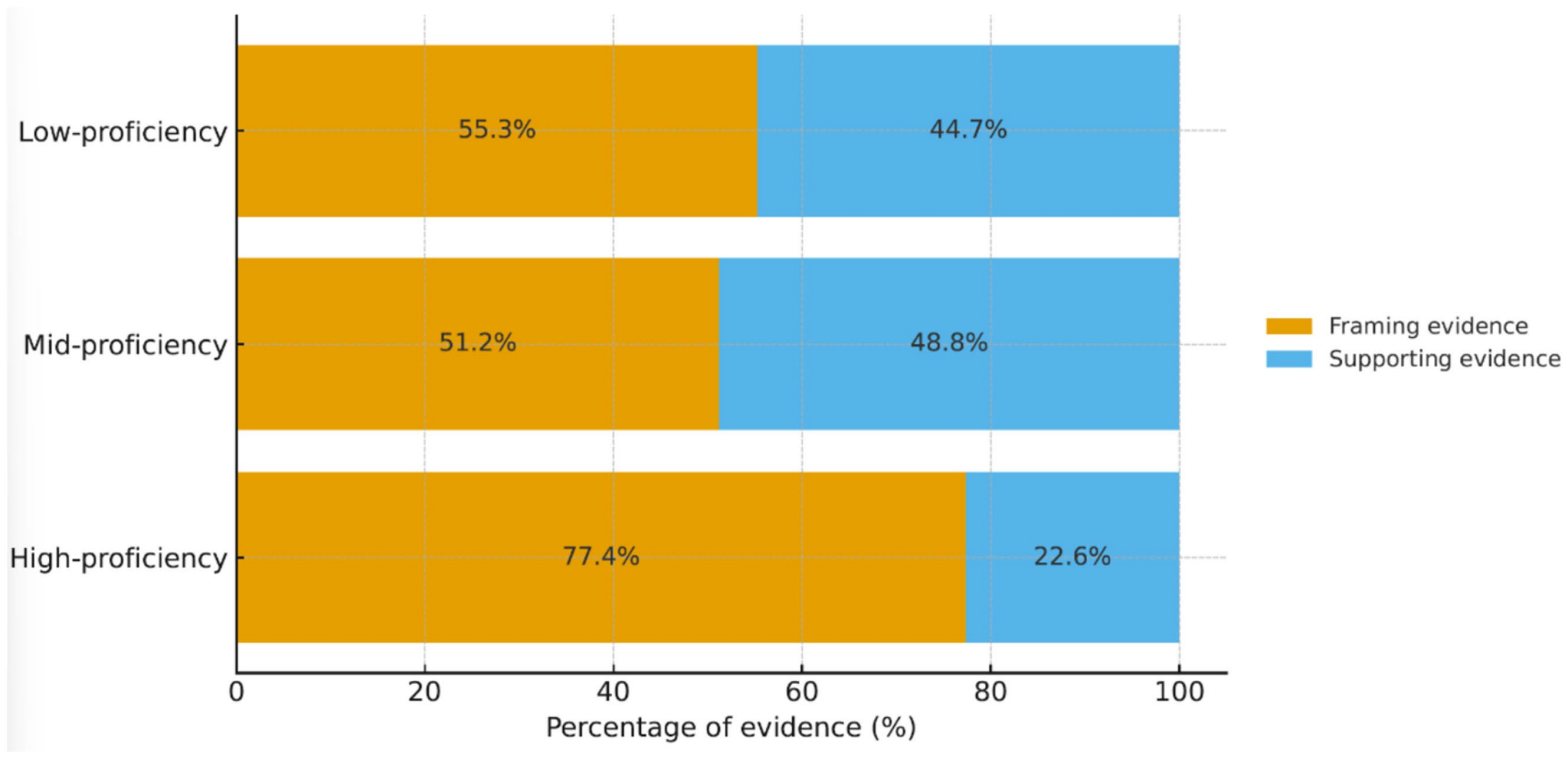
Proportional distribution of different evidence types across score ranges. Stacked bars show within-group percentages (framing + supporting = 100%). High-proficiency writers rely far more on framing evidence (77.4%) than supporting evidence (22.6%), whereas mid- and low-proficiency groups are comparatively balanced (≈51–55% vs. 45–49%). Note: Framing evidence = claim-integrated reasoning that structures the argument; Supporting evidence = externalized support (e.g., examples, statistics, expert citations).

Table 3 reports the count distribution of supporting versus framing evidence across the three groups. All groups are dominated by framing evidence, with the largest gap in the high-scoring group (216 vs. 63). The mid-scoring group shows the highest overall production and the most balanced mix of the two types (221 vs. 211). The low-scoring group has the fewest instances of both (119 and 96), indicating an overall insufficiency in evidence generation (Figure 2).

**Table 3:** Distribution of framing vs. supporting evidence across proficiency groups.

Group	Total evidence (*n*)	Framing evidence, *n* (%)	Supporting evidence, *n* (%)
High-proficiency	279	216 (77.4)	63 (22.6)
Mid-proficiency	432	221 (51.2)	211 (48.8)
Low-proficiency	215	119 (55.3)	96 (44.7)

Percentages are within-group. Framing evidence denotes claim-integrated reasoning that structures the argument; supporting evidence denotes externalized support such as examples, statistics, and expert citations.

### 4.2 Distribution of evidence types across proficiency levels among university students and their explanatory power for writing scores

**Figure 3:**
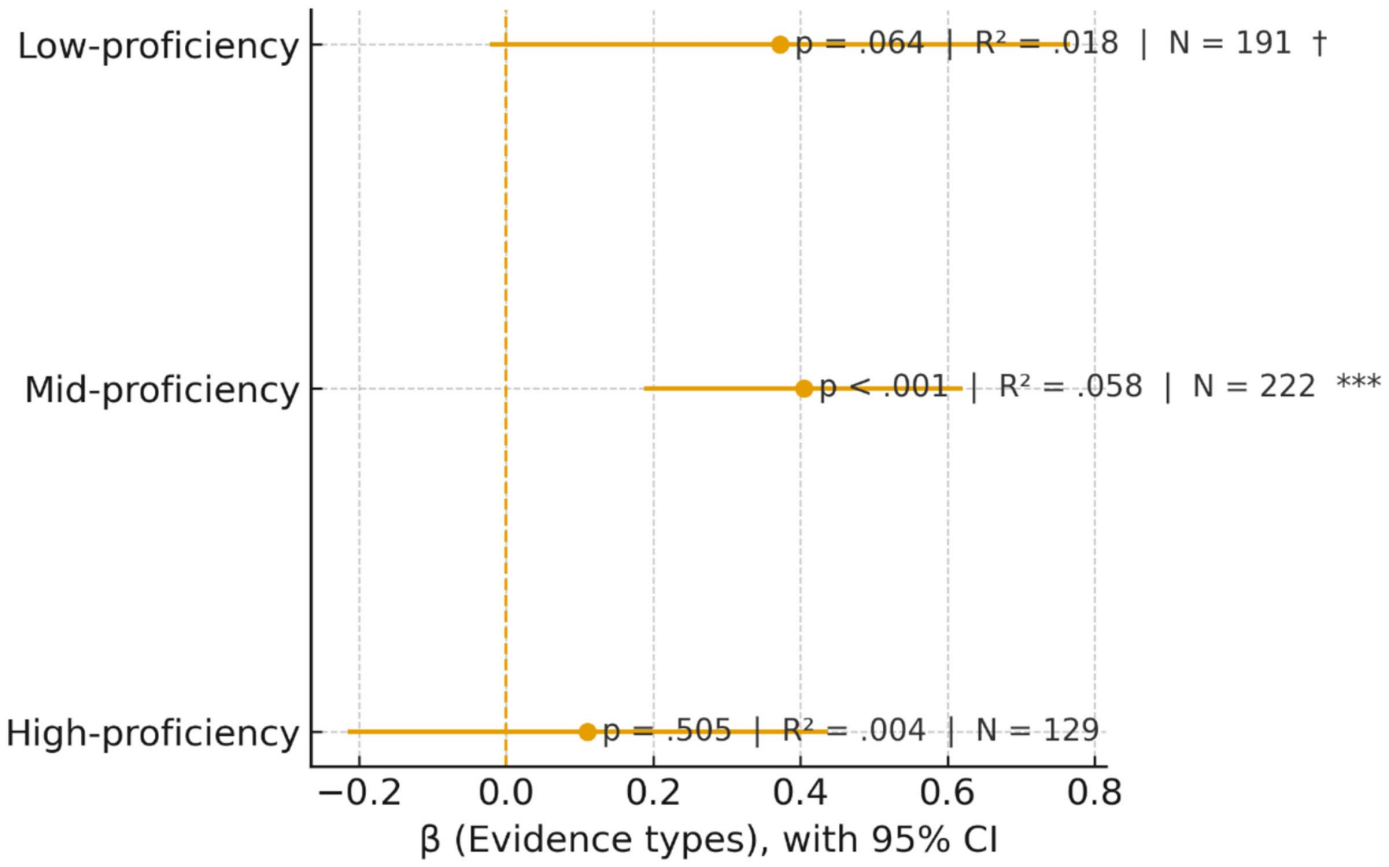
EviT (Evidence types) predicts writing scores: a proficiency-wise forest plot.

Figure 3 shows the regression coefficients (β) of writing score on evidence types (EviT) for each proficiency group, with 95% confidence intervals. The vertical dashed line at zero marks β = 0 (no effect), and annotations report the *p* values, R^2^, and sample sizes. Significance codes: † *p* < 0.10, * *p* < 0.05, ** *p* < 0.01, *** *p* < 0.001.

### 4.3 Writing examples across proficiency groups

#### 4.3.1 Diversity of evidence—demonstrating specialized knowledge and authority

*In my view, marine parks are unnecessary if they cause harm to creatures. For example, at SeaWorld in Florida, killer whales live in small tanks. This makes them unhappy and sometimes dangerous. The film Blackfish shows these problems (Evidence–Supporting–Example). A study in Marine Mammal Science (2018) says dolphins in tanks live only about 12 years, but in the wild they can live more than 30 years (Evidence–Supporting–Data). (High-Proficiency Group–No. 14).*

The passage triangulates a concrete case (SeaWorld), documentary material (*Blackfish*), and peer-reviewed data (*Marine Mammal Science*, 2018), yielding diverse types of evidence from credible sources. The argumentative trajectory advances in a layered sequence—case example → documentary revelations → statistical data—balancing affective resonance with verifiable quantitative support. All evidence converges on the central claim that captivity causes harm and shortens lifespan, producing a mutually corroborating effect that substantially strengthens the argument’s persuasive force. Explicit attribution of sources and years evidences sound scholarly search and citation practices, underscoring the high-scoring writer’s professional competence and authority.

#### 4.3.2 Integration of argumentative function and evidence: coordinating multiple argumentative elements

*Marine parks can support scientific research by offering controlled environments to study marine life (Reason). Marine parks function as research hubs, offering controlled settings that enable precise studies of marine life (Evidence–Framing). For example, dolphin communication has been systematically recorded and analyzed in such facilities, yielding insights hard to obtain in the wild (Evidence–Supporting–Example). Some say controlled settings change animals’ behavior and do not show real ocean life (Counterargument). But they still give basic data and repeatable tests that help guide research in the wild (Rebuttal). (High-Proficiency Group–No. 33).*

The text unfolds in a closed-loop sequence—“reason → framing evidence → supporting evidence (examples) → counter-position → rebuttal”—demonstrating strong functional alignment between argumentative purposes and types of evidence. It first links the reason to “controlled environments/research hubs” to construct and consolidate the inferential scaffold of the subclaim; it then operationalizes this at the micro level with verifiable evidence (e.g., systematic records and analyses of dolphin communication), thereby establishing a “structuring–instantiating” division of evidential labor. Next, it introduces the counter-position that controlled settings distort behavior and offers a targeted rebuttal by arguing that foundational datasets and repeatable tests can feed back into field research, showcasing the anticipatory and responsive capacities of dialogic argumentation. Overall, the structure is rigorous, evidence consistently serves the claim, and the reasoning achieves a logical extrapolation from controlled contexts to field applications, thereby enhancing the argument’s external validity and overall persuasiveness.

#### 4.3.3 Characteristics of the medium-proficiency group—explaining reasons in a step-by-step manner

*First, what animals really want is freedom, and they may dislike being fed and watched like toys (Reason 1). They hold an enthusiasm for the sea and hope to stay with their families (Evidence 1—Framing). In addition, some merchants do not care about the lives of marine animals; what they prefer is only money (Reason 2). For example, some parks keep sick dolphins performing because they want to sell more tickets (Evidence 2—Supporting: Example). Finally, people should not break the balance with marine animals (Reason 3). If we do such a stupid thing, we will finally get the punishment from nature (Evidence 3—Framing). (Medium-Proficiency Group–No. 101).*

The passage is characterized by orderly organization: the author sequences three reasons with “First–In addition–Finally.” Each reason first provides framing (values and principles such as freedom and ecological balance) and then substantiates it with examples (e.g., sick dolphins being forced to perform), producing a coordinated “stance first, then evidence” exposition. Normative judgments and verifiable facts—“soft” and “hard”—work in concert, yielding a tidy structure, smooth logic, and ample information; readers can follow readily, and the overall persuasiveness is strengthened.

#### 4.3.4 Characteristics of the low-proficiency group: lack of evidence and creativity

*Second, animals may get sick (Reason). Animals may feel unwell (Evidence 1—Framing). Animals could lose their health (Evidence 2—Framing). (Low-Proficiency Group–No. 64).*

The passage repeatedly paraphrases “animals may get sick” as “feel unwell/lose their health,” which constitutes a framing restatement without adding new information. Lacking supporting evidence (e.g., concrete cases, data, or authoritative sources), the argument becomes self-referential and non-verifiable. The expression is uniform and repetitive, with little situational specificity or causal chaining, revealing evidence scarcity and limited originality; consequently, its persuasive force is weak.

## 5 Discussion

### 5.1 Layered characteristics and developmental trajectory of evidence types

From the low- to the high-scoring group, a trajectory emerges from “frame-reliant → semi-integrated → diversely supported.” Low scorers tend to remain within internal explanations and synonymous extensions of the reason sentence, with insufficient external supporting evidence, making it difficult to form a closed evidence–reasoning–claim loop (Vilar Weber and Tolchinsky, 2022; Du and List, 2021; McCann, 1989). Textually, low scorers often combine generalized assertions with affective/common-sense exposition; source attribution is unstable, evidence density is low, and the functional fit with the claim is weak. This aligns with the genre-developmental path described by Vilar Weber and Tolchinsky (2022), which moves from “assertion-centered” writing toward “explanatory/evidence-led” discourse. The observation also resonates with recent instructional and assessment orientations in the field—shifting attention from “whether/how much is cited” to “how evidence is selected, interpreted, and functionally integrated with claims,” with corresponding emphasis on purposeful source use and integration quality in both scoring dimensions and teaching scaffolds (Chuang and Yan, 2023; Amini Farsani et al., 2025).

The mid-scoring group exhibits semi-integration: they can, to some extent, connect explanatory information with concrete evidence, but the functional alignment of evidence and reasons—and the depth/precision of cross-source integration—remain limited. Writing is often led by framing reasons and supplemented with scattered facts or quotations; there is relatively little coordination of source credibility, evidence appropriateness, or conflicting information, and counter-positions/rebuttals tend to remain at a general level. This profile accords with genre-development findings: the full maturation of explanatory and evidential components typically appears at more advanced stages (Vilar Weber and Tolchinsky, 2022). Multiple-document studies likewise show that mid-level learners gravitate toward surface processing (excerpts, paraphrase) rather than deep processing (evaluation, synthesis, transformative recomposition), making high-quality evidence–reason linkages harder to achieve (Du and List, 2021; List and Alexander, 2017; List, 2020). Their selection and integration are further influenced by cues such as topic familiarity, authorial authority, and content relevance, resulting in unstable integration quality (Bråten et al., 2018; Kullberg et al., 2023). In L2 assessment contexts, raters do perceive the functional use and integration quality of sources and adjust argumentation scores accordingly; the mid group’s “semi-integration” thus corresponds to mid-level performance (Chuang and Yan, 2023; Chuang, 2025).

High scorers flexibly and fully mobilize external supporting evidence (examples, data, authoritative quotations, expert views) and configure these with reasons into “example/data + reasoning” alignments, thereby enhancing testability and persuasiveness (Packer and Timpane, 1997; Vilar Weber and Tolchinsky, 2022; Abdollahzadeh et al., 2017). Their texts more often show explicit handling of source credibility, anticipation/delimitation of counter-examples, and functionally aligned expressions following cross-source synthesis. This profile matches the converging source-integration → argument quality → rating evidence: when evidence is functionally aligned with claims and well integrated, it is typically accompanied by stronger argumentative effectiveness and more favorable rater judgments on the “argumentation” dimension (Chuang and Yan, 2023; Chuang, 2025; Sandoval and Millwood, 2005).

### 5.2 Mechanisms linking evidence quantity, type, and functional integration to writing quality

Overall, high scorers not only outperform in evidence quantity and type diversity, but also more effectively align external evidence—examples, data, authoritative quotations, and literature-based claims—with reasons in structured “example/data + reasoning” configurations, and more frequently incorporate counter-positions and rebuttals to enhance testability and persuasiveness (Packer and Timpane, 1997; Qin and Karabacak, 2010; Stapleton and Wu, 2015; Zohar and Nemet, 2002; Brem and Rips, 2000). The observable mechanism is: first, screen task-relevant evidence that can be *interfaced* with the claim; next, bridge evidence and reason via explicit or implicit warrants; finally, consolidate boundary conditions through qualification and rebuttal, thereby improving argumentative robustness and discriminability.

By contrast, while the mid group may appear balanced on the surface (framing vs. supporting evidence), the depth of integration and contextualized reasoning is often insufficient. Hence, merely having evidence or having “more of it” does not guarantee high scores; the critical factor is functional alignment and organization of evidence with the claim/task purpose (Du and List, 2021; List and Alexander, 2017; Strømsø and Bråten, 2014; Nussbaum et al., 2019). Common risks include list-like stacking of evidence without bridging discourse, inadequate handling of conflicts across heterogeneous sources, and failure to “return” to the reason/claim to complete the logical loop at the end of a paragraph.

Low scorers commonly show weaker structural control and sparse evidence, resembling length extension rather than argument deepening. This profile accords with NAEP characterizations of weaker texts and with findings on Chinese EFL writers’ challenges in evidence quality and logical support (National Center for Education Statistics, 2012; Stapleton and Wu, 2015). Moreover, constraints in linguistic resources (syntactic/lexical complexity) suppress effective evidence–reasoning linkages, affecting the explicitness and precision of warrants—a background factor operating across proficiency levels (Atak and Saricaoglu, 2021, Saricaoglu and Atak, 2022).

Synthesizing prior work with the present statistics, and controlling for language proficiency, the functional integration quality of evidence (selection–explanation–alignment) predicts writing proficiency better than sheer quantity (Chuang and Yan, 2023; Chuang, 2025; Sandoval and Millwood, 2005). Accordingly, understanding writing quality should prioritize process-level indicators of “how evidence becomes reason” (e.g., evidence density and explicitness of alignment) rather than “whether/how much”; this perspective also supports the pedagogical and assessment shift.

## 6 Implications

Based on this study and related evidence, instructional and assessment priorities should move from “whether/how much evidence” to the functional fit between evidence and the reasons it is meant to support. Concretely, the writing process can be organized into a three-step Claim–Frame–Evidence (C–F–E) sequence: first, use one or two sentences to set evaluation criteria and causal chains (the *frame*); then provide verifiable support (data, authority, cases); and finally make the warrant explicit—why this evidence substantiates this reason—so that the evidence–reasoning–claim loop is visible (Chuang and Yan, 2023; Cottrell, 2011). Operationally, “micro-tasks + sentence-level scaffolds” can lower the barrier to composing warrants (e.g., a template such as “Because X instantiates criterion Y, it suffices to support Z”), coupled with 1–2 rounds of in-class peer review to check the visibility of functional alignment (Chuang and Yan, 2023).

Implementation should be tiered. For low–mid groups, draw on multi-document learning evidence to prioritize “search–evaluate–integrate,” source attribution/paraphrase skills, and explicit checks on the accuracy of explanation and the *purpose/mode* of integration; this reduces surface excerpting/synonym paraphrase and improves functional integration (Wiley and Voss, 1999; Chuang and Yan, 2023; Judd et al., 2006; Nussbaum et al., 2019). Building on this, adopt structured formative feedback combining rubrics and strong exemplars to stabilize and transfer deeper elements such as rebuttal/qualification (Peltzer et al., 2024a, 2024b; Peltzer et al., 2025; Burnell et al., 2023; Panadero, 2025; Bin Dahmash, 2025; Balwanz-Emmel, 1989). Where appropriate, introduce dialogic/collaborative argumentation (peer review and group revision), using the cycle “frame first → support next → revise the warrant” to tighten the match between evidence and argumentative structure (He and Du, 2024). For high scorers, while maintaining evidence diversity, further optimize the precise articulation of evidence–reasoning–claim; guard against “piling evidence with weak reasoning” by requiring a one-sentence warrant at paragraph ends and a short checklist for self-auditing verifiability/relevance/sufficiency (Chuang and Yan, 2023).

## 7 Limitations and future directions

### 7.1 Limitations

First, the writing task and genre were relatively uniform and constrained by a “text budget”: a single prompt and timed short essay (30 min; 120–180 words) under CET-4 conditions. The short time and limited length objectively restrict argumentative elaboration and multi-source integration, easily producing a ceiling effect on observable evidence diversity within a single text, which may underestimate learners’ true capacities in complex reasoning and multi-source integration (Wiley and Voss, 1999).

This is likely due more to task structure and length limitations than to learners’ “upper bound.” Although we enhanced identification consistency under short-text conditions through rater training, priority rules for conflict resolution, and adjudication, the assessed richness of evidence types may still be affected by the text-budget constraint.

Second, the study is cross-sectional, revealing associations rather than causal relations. Although groupwise regression showed significant effects in the mid group, the modest R^2^ suggests additional factors (e.g., linguistic complexity, planning/revision processes) not included here and best controlled with process data in future research.

Third, for operational feasibility we dichotomized evidence into *framing/supporting*; however, boundary cases (e.g., analogy, common-sense exposition) call for finer decision rules and more raters to improve agreement and to test the discriminant validity of subtypes (Du and List, 2021; Vilar Weber and Tolchinsky, 2022).

Fourth, the sample consists of Chinese first-year EFL students and centers on the argumentative genre; generalization to other grade levels, disciplinary genres, or L1 backgrounds should be cautious, with attention to topic familiarity and task constraints that shape evidence selection.

### 7.2 Future directions

First, conduct longitudinal and intervention studies: track training organized around “frame → support → rebut/qualify” over a semester or year; compare the gains from structured formative support (rubrics + exemplars) versus dialogic mediation/dynamic assessment; use multiple measurement points to examine transfer and retention; and record process indicators (e.g., time allocation to planning–drafting–revision, proportion of paragraphs with explicit warrants) to model mechanisms of growth (Chuang and Yan, 2023; He and Du, 2024; Peltzer et al., 2024a, 2024b; Peltzer et al., 2025).

Second, refine assessment tools and explore human–AI collaboration: convert the study’s coding indices into computable features (e.g., *framing-sentence density*, *explicit evidence–reason alignment rate*, *proportion of attributed claims*); develop a human–AI prototype for scoring/diagnosis; and systematically test structural, incremental, and predictive validity, along with cross-task/genre consistency and stability, to enhance usability and scalability (Chuang and Yan, 2023).

Third, expand task conditions to mitigate the “text-budget” constraint: introduce longer essays and extended time windows (e.g., TEM-4 tasks for English majors), and design multi-source, material-driven argumentative tasks or take-home writing to allow broader argumentative development and a higher observable ceiling for evidence types. Compare, in a systematic way, evidence subtype distributions, combination patterns, and predictive validity across tasks (short vs. extended), cohorts (general vs. disciplinary English), and text lengths, thereby assessing learners’ evidence use in authentic academic contexts and calibrating tiered teaching and assessment plans.

## 8 Conclusion

Taking the functional perspective on evidence, this study proposed and tested a two-level framework of framing vs. supporting evidence, and described their distributions across proficiency groups. Quantitatively, evidence type significantly predicted writing quality only in the mid group, but not in the high/low groups. This supports a functional threshold account: once a text surpasses a minimally effective configuration, further quality gains depend more on warrant precision and functional alignment between evidence and claims than on simply adding more evidence items (Chuang and Yan, 2023; Plakans and Gebril, 2013; Brem and Rips, 2000). Hence, assessing “how integration happens” explains quality variation better than assessing “whether citation occurs,” and it helps clarify the mid group’s “sensitivity window.”

The qualitative analysis further revealed a division-of-labor mechanism in effective texts: framing evidence handles problem scoping, evaluative criteria, and causal/conditional chains, while supporting evidence (examples, data, attributed claims) validates and “stress-tests” those chains, ensuring functional alignment among claim, reason, and evidence. This linkage is consistent with classical argumentation models (Toulmin, 1958; Packer and Timpane, 1997; Williams and Colomb, 2007) and with L2 observations that purposeful source use is more persuasive (Chuang and Yan, 2023). By contrast, low-proficiency texts frequently display an imbalance of “assertion-dense, evidence-sparse,” echoing large-scale assessments of weaker argumentative writing (National Center for Education Statistics, 2012).

Pedagogically, evidence points to a more efficient pathway than dispersed marginal comments: explicit scaffolds and targeted exemplars/rubrics more reliably elicit and stabilize deeper elements such as rebuttal and qualification (Peltzer et al., 2024a, 2024b; Peltzer et al., 2025; Burnell et al., 2023; Panadero, 2025), while classroom traditions of modeling with exemplars/templates offer actionable evidence (Balwanz-Emmel, 1989). Differences in advanced evidence use among EFL students also indicate that interventions should be developmentally staged (Abdollahzadeh et al., 2017). Accordingly, instruction can advance on three fronts: (a) staged training that differentiates and progressively integrates framing moves (scoping, standard-setting, causal linking) and supporting moves (examples/data/authority); (b) requiring writers to make the warrant explicit, articulating the testable link between evidence and reason; and (c) embedding functional alignment as a core dimension in formative assessment and feedback (Chuang and Yan, 2023).

Finally, the current model’s explanatory power remains limited, cautioning against over-attributing quality variance to evidence features alone. Future research should test, in longitudinal/process-tracing designs, the transfer and durability of explicit “frame–support–rebut/qualify” training across tasks and genres; methodologically, the coding indices here can be embedded in mixed automatic–human assessment pipelines for both feature engineering and human calibration/alignment (Peltzer et al., 2024a, 2024b; Peltzer et al., 2025). This approach promises greater consistency and efficiency in classroom evaluation and research measurement while preserving academic interpretability.

## Data Availability

The datasets presented in this article are not readily available because they are private and cannot be shared publicly. Further inquiries should be directed to Rui Yang, yangrui@ahstu.edu.cn.
