# Correction: Framing vs. supporting evidence in L2 argumentative writing: a mixed-methods study of Chinese EFL learners

**DOI:** 10.3389/fpsyg.2026.1782179

**Published:** 2026-01-28

**Authors:** Rui Yang

**Affiliations:** 1 School of Foreign Languages, Anhui Science and Technology University, Chuzhou, Anhui, China; 2 Key Laboratory of Human–AI Collaborative Translation and Education, Anhui Science and Technology University, Chuzhou, Anhui, China

**Keywords:** L2 argumentative writing, evidence use, framing evidence, supporting evidence, proficiency differences, source-based writing, mixed methods, assessment

There was a mistake in Figure 1 as published. The *x*-axis was erroneously cut off. The corrected Figure 1 appears below:

**Figure 1:**
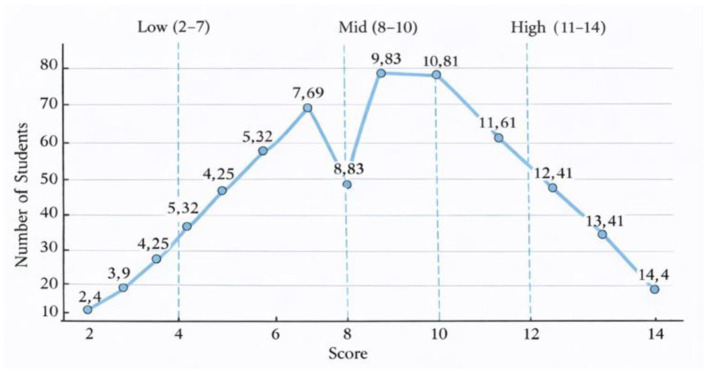
Distribution of English argumentative essay scores.

The original version of this article has been updated.

